# Preventive herd management practices and their effect on lamb mortality in Ethiopia

**DOI:** 10.1007/s11250-022-03361-x

**Published:** 2023-01-19

**Authors:** E. Genfors, U. Magnusson, M. M. Moliso, B. Wieland, U. König, G. S. Hallenberg, R. Båge

**Affiliations:** 1 Department of Clinical Sciences, Faculty of Veterinary Medicine and Animal Science, Swedish University of Agriculture Agricultural University (SLU), Box 7054, 750 07 Uppsala, Sweden; 2 International Livestock Research Institute (ILRI), Addis Ababa, Ethiopia; 3 Present Address: Institute of Virology and Immunology, Mittelhaeusern, Switzerland; 4 Present Address: Department of Infectious Diseases and Pathobiology, Vetsuisse Faculty, University of Bern, Bern, Switzerland; 5 Farm and Animal Health Organisation, Uppsala, Sweden; 6 Present Address: Public Health Agency of Sweden, Stockholm, Sweden

**Keywords:** Lamb mortality, Preventive management practices, Sheep herd health, Ethiopia

## Abstract

According to previous studies, lamb mortality is high in the Ethiopian highlands. The present study aims to evaluate the execution of preventive sheep herd health management practices with respect to if, and how, such practices are linked to occurrence of lamb mortality. Interviews were performed with 74 sheep-owning households participating in a capacity development program on livestock and 69 households not participating in such program. To evaluate the impact of combinations of performed practices, a scoring system was developed—the households retrieved a higher score the more desired routines were accomplished. To identify which practices had the highest impact on lamb mortality, a similar score was calculated for each phase of the sheep reproductive year, creating sub-scores for each phase. The results showed a significant (*p* < 0.05) negative correlation between the total number of performed practices and occurrence of lamb mortality, indicating a lower occurrence of lamb mortality the more desired practices implemented. Further analysis of sub-scores showed significant (*p *< 0.05) negative correlations between a higher number of performed desired practices during gestation period and during lambing. Conclusively, the study indicates that preventive herd management routines are beneficial for lamb survival, foremost when enforced during the gestation period and around lambing—hence, this is where to focus future interventions.

**Supplementary Information:**

The online version contains supplementary material available at 10.1007/s11250-022-03361-x.

## Introduction

Ethiopia has the largest number of small ruminants in Sub-Saharan Africa, including about 40 million sheep (CSA 2020). Livestock keeping is essential for Ethiopian smallholder farming households and an important source of income all over Ethiopia (Fentie et al. 2016). Several studies show that lamb mortality in Ethiopia is high, at times reaching 50%, and thus becomes one of the main restraints in sheep productivity (Mukasa-Mugerwa et al. 2000; Fentie et. al 2016; Ayele and Urge 2018). The main causes of lamb mortality are multifactorial with high endemicity of respiratory and gastrointestinal diseases (Mukasa-Mugerwa et al. 2000; Tibbo et al. 2003; Fentie et al. 2016); however, studies on detailed preventive management practices to reduce impact of these diseases are lacking (Tibbo et al. 2003; Fentie et al. 2016).

Preventive herd health management aims to improve the overall sheep health by mitigating disease, including subclinical diseases. The subclinical diseases, regardless of origin (infectious agents, deficiencies, etc.), are main factors for animals performing and producing below capacity. The nature of such diseases often makes them go undetected, which in turn might result in extensive production losses (Kyriazakis et al. 1994; Sargison et al. 1997; West et al. 2009). With the use of disease preventive management strategies, one can increase the farmer’s profit and, additionally, improve animal welfare and use natural resources more efficiently (Roger 2008; Rojas-Downing et al. 2017).

Lamb survival has been correlated to the status and management of the ewe throughout her reproductive cycle, as well as during gestation and lactation, and hence, is linked to the herd management practices. Previous studies have suggested, for example supplementary feeding of ewes in poor condition before mating, planned mating to prevent unnecessary strenuous environmental conditions at birth, proper housing with good hygiene at lambing, and monitoring and support of weak lambs—all to increase the survivability of the lamb (Tibbo et al. 2001; Binns et al. 2002; Holmøy et al. 2012).

The current study was conducted in the Amhara region in the Ethiopian highlands. The region is a densely populated area with a high ratio of small ruminants per household, as sheep are the main source of livelihood—despite limited animal feed and land area (Gizaw et al. 2010; Tilahun and Schmidt 2012). The overall objective of the study was to examine present management practices performed by smallholder sheep farmers, evaluate the correlation between such practices and lamb mortality, as well as identify opportunities to increase lamb survivability in the area.

## Materials and methods

### Study area and data collection

A study based on face-to-face interviews was performed during September through November 2019 in Menz area, Amhara region, Ethiopia. The target population for the study was rural smallholder sheep farmers from two types of villages, those which had participated in previous research or capacity-building projects run by CGIAR Research Program on Livestock, or affiliated organisations (so-called intervention villages) and those which had not participated in such projects (non-intervention villages). The aim was to interview a total of 150 farmers.

Households were included if they had at least one newborn lamb (< 7 days old). Information about newborn lambs was collected by local animal health workers who had established contact with the farmers. If there were more households with newborn lambs in the area than the research team had time to interview in 1 day, the ones with the oldest lambs were visited first followed by the ones in nearby perimeter.

### Questionnaire

The interviews followed a questionnaire (enclosed as attachment 1), which aimed to evaluate sheep flock practices and was developed in collaboration with Swedish and Ethiopian sheep researchers and experts. The questionnaire consisted of 97 questions designed to incorporate all aspects of sheep keeping and categorised into phases of the sheep reproductive cycle, summarised in Table 1.

**Table 1:** Description of the phases of the sheep year. For all questions, see questionnaire (attachment 1)

Phase	Description of phase
1. Household details	General information about the household such as main caretaker, education level, and previous experience of sheep keeping
2. General flock health and practices	General information about the flock health such as disease occurrence, as well as general preventive measures including, e.g. isolation, vaccination, and deworming routines
3. Lamb health over the past year	Information about general lamb health, including number of born and dead lambs during the past year and possible symptoms before lamb mortality occurred
4. Mating period	Practices for, e.g. heat detection and choosing of what ram and ewe to mate. Phase stretches from when the ewe is allowed to mate until she is pregnant
5. Pregnancy	Practices for pregnancy detection and special measures taken during gestation period. Phase stretches from fertilisation of the egg until the very end of the gestation period
6. Time around lambing	Practices for detection of signs of delivery, practices in assistance at lambing. Phase stretches from a few days before delivery until the moment the lamb is born
7. Neonatal period (< 7 days)	Practices regarding lamb colostral intake and monitoring, as well as monitoring of the newborn lamb and the ewe. Phase stretches from the moment the lamb is born until it reaches the age of 7 days
8. Later lamb life (7 days < 6 months)	Practices regarding weaning, grazing, and special feed and treatments for the lamb. Phase stretches from the lamb is 8 days until 6 months of age

The questionnaire contained a mixture between close- and open-ended questions, including a number of checkbox questions. For each checkbox question, the interviewees were presented with the option “other” with a chance to elaborate. Some questions were designed in a skip pattern—these were questions where further description of actions was sought and, in other words, irrelevant to ask the households who did not execute the action at hand. The questions that all households answered will henceforth be referred to as level one questions. If interviewees provided information supplementary of the questions, it was noted and saved as informal information.

The questionnaire was pre-tested on farmers in the intended research area to assess quality and feasibility before the study commenced. Interviews were carried out by the first author together with an Amharic translator with knowledge of veterinary medicine, the same translator throughout the study.

### Data management and statistical analysis

The questionnaire was created in, and collected data was entered into Epi Info™ 7.2.3.1 software, and, thereafter, exported to and analysed in Microsoft Excel and the statistical software R (R Core Team 2020) for analysis. As some questions had multiple possible answers, the data was transposed into binary data. Fisher’s exact test (chosen due to multiple variables with few observations) and logistic regression were performed on binary and continuous explanatory variables, respectively, to evaluate the correlation of specific practices to the binary outcome “occurrence of lamb mortality during the past year”. Any mortality occurrence of an ovine < 6 months was registered as lamb mortality. Backward stepwise logistic regression was executed on the 44 level one questions—as these were the questions answered by all households—to see if there were any combinations of practices that could be associated with occurrence of lamb mortality.

The next analysis aimed to evaluate possible correlations between mortality rates and the number of desired practices that was carried out by the households. This required an establishment of which practices were desired (defined as “believed to be protective for lamb health”), and for this cause, every question and answer were evaluated by a group of Ethiopian and Swedish sheep experts. Questions that were not purely investigating the household’s practices, but instead examined contextual covariates (such as weather circumstances, farmer perceptions, and education level), were excluded from this analysis.

Every desired practice—including refrainment from carrying out an undesired one—was assigned a total value of 4 points. The skip pattern questions with multiple levels of questions could still only be worth a maximum of 4 points, to not over-value details of management practices. The total sum of the points generated an individual index score (from now on referred to as score) for each household, where the more desired practices executed, the higher the household’s score. There were 27 4-point questions, creating a maximum score of 108. The score was thereafter included in a logistic regression model to evaluate relationship to occurrence of lamb mortality.

To evaluate the impact of the executed practices in more detail, a division of the desired practices was made into the different phases of the sheep reproductive cycle based on the design of the questionnaire (see Table 4). These were about the household and general flock practices (phases 1, 2, and 3 combined), mating period (phase 4), pregnancy (phase 5), time around lambing (phase 6), neonatal period (phase 7), and later lamb life (phase 8). The same scoring system was used to calculate a subtotal score for each phase, and logistic regression was run on the subtotal scores to individually evaluate each section and its relationship to occurrence of lamb mortality. All phases were also run in a backward stepwise regression to evaluate which phases were more strongly correlated to occurrence of lamb mortality.

The same scoring system was again used to evaluate the presence of desired practices in intervention versus non-intervention villages in a two-sided *t*-test.

## Results

For this study, 143 households were interviewed of which 74 were intervention villagers and 69 were non-intervention villagers. The average sheep flock had 21 sheep (ranging between 5 and 92), of which 12 (2–53) ewes, 3 (0–14) rams, and 6 (1–25) lambs. The sheep were of local breed, generally kept in small mixed crop-livestock systems, in small- to medium-sized flocks (5–92 individuals).

The participating households reported a total of 1476 lambs born during the past year, of which 116 died. This equals a lamb mortality rate of 8% (average 0.81 lamb deaths and median 0 lamb deaths per farm). These deaths occurred on 56 of the 143 farms (39% of the farms), generating a lamb mortality of 22% on the affected farms (average 2 lamb deaths per farm). Considering the number of days per age category, the highest mortality rate was reported to occur during the neonatal period (see Table 2).

**Table 2:** Lamb age at death and average number of lamb deaths per day in each age category

Age of lamb at death	Number of lamb deaths occurring at this age (*n*)	Percentage of total lamb deaths occurring at this age (%)		Average lamb deaths per day (*n*)
0 d*	12	10	12	
1–8 d	21	18	3	
9–30 d	24	21	1.1	
30–90 d	27	23	0.5	
91–180 d	29	25	0.3	
After weaning	3	3	-	
Total	116	100	-	

^*^Stillbirths not specified

The reported causes of death were as follows, listed from most to least common (number of reported cases): starvation (12), general weakness (11), unknown (9), respiratory distress (8), gastrointestinal diseases (7), sudden death (6), trauma (5), neurological disease (4), other reasons (4), and abnormalities (3). Lamb mortality was most common during the dry season (January–June) where 58% of the lamb deaths occurred.

### Single factors associated with lamb mortality

Ten out of 273 explanatory variables were found to have a significant correlation to the binary outcome of lamb mortality (Table 3). The significant variables were from all eight sections of the questionnaire; eight were classified as practices and two as contextual covariates.

**Table 3:** Univariate analysis: significant (*p* < 0.05) practices performed (according to household) associated with occurrence of lamb mortality

Variable	Odds ratio	Positive or negative association with lamb survivability	Phase of sheep reproductive cycle*	Practice (Yes/No)
Farmer has finished junior secondary school	0.68	+	1	No
Farmer has been educated via old school system	4.1	−	1	No
Farmer does not restrict contact between own sheep and other sheep	2.65	−	2	Yes
Farmer checks ewe body condition score in other ways than using eye measurement or palpation of ewe	∞	−	4	Yes
Farmer states that a sign for the ewe being pregnant is that she weans of her old lamb	0.3	+	5	Yes
Farmer regroups the sheep when the ewe is known to be pregnant	0.43	+	5	Yes
Farmer uses plastic gloves if assisting the ewe at delivery	0.21	+	6	Yes
Farmer makes sure the lamb gets sufficient amounts of colostrum	0.26	+	7	Yes
Farmer guides the lamb to the teat in the event that mismothering occurs	0.53	+	7	Yes
Farmer states that the lamb weans off at 6 months or earlier	0.46	+	8	Yes

^*^See Table 1 for description of phases

In the stepwise regression method on level one questions, the variables best explaining the binary lamb mortality outcome were the ones presented in Table 4, where one variable (“Farmer stated that he/she manages the pregnant ewes differently than the non-pregnant ewes”) was significant (*p* = 0.03).

**Table 4:** Multivariate analysis: level one variables most closely associated with occurrence of lamb mortality

Variable	*P*-value	Practice (Yes/No)
If the household belonged to an intervention village	0.151	No
Household has experienced health issues within the herd during the past year	0.054	No
Household knows on beforehand how many lambs the ewe is carrying	0.125	Yes
Household manages the pregnant ewes differently than the non-pregnant ewes	0.032	Yes
Farmer knows on beforehand when the ewe is expected to lamb	0.147	Yes

### Combinations of variables associated with lamb mortality

Household’s total practice score was significantly and negatively correlated with occurrence of lamb mortality (*p* = 0.036), indicating that an increase in routine score gave a decreased occurrence of lamb mortality. Logistic regression on the sub-scores in each phase resulted in significant negative correlations between occurrence of lamb mortality and management during phase pregnancy (*p* = 0.0397) as well as during phase time of lambing (*p* = 0.0489)—again suggesting that performing desired practices during these phases was associated with lower occurrence of lamb mortality.

### Comparison of execution of practices between intervention and non-intervention villages

There was no significant difference in occurrence of lamb mortality depending on whether the household belonged to an intervention village or not. Neither was there any significant differences between the two types of villages when comparing total score means using a two-sided *t*-test. However, when comparing the sub-score means in each phase (see Table 4), there were significant differences between the village types during phase mating (“Questionnaire”, *p* = 0.00725), phase pregnancy (“Data management and statistical analysis”, *p* = 0.0182), and phase early lamb life (“Combinations of variables associated with lamb mortality”, *p* = 0.0394), where intervention villages had higher score means in all three phases.

### Data availability statement

The datasets generated and analysed during the current study can be retrieved from the Swedish National Data repository at 10.5878/arqm-vq14.

## Discussion

Preventive sheep flock management practices are beneficial for disease mitigation, consecutively increasing sheep productivity, welfare, and economical benefit for the owner (Roger 2008; Rojas-Downing et al. 2017). Preventive herd management includes treatments such as vaccination and deworming and also incorporates actions such as mating practices, feeding practices, assistance during lambing, and close supervision of ewe and lamb during the early lamb life. The use of preventive herd management has previously been linked to increased lamb survival (Binns et al. 2002; Tibbo et al. 2003; Holmøy et al. 2012). Earlier studies from Ethiopia present a high prevalence of lamb mortality (Mukasa-Mugerwa et al. 2000; Fentie et al. 2016; Ayele and Urge 2018); however, studies on and application of preventive herd management practices in the country have been lacking (Tibbo et al. 2003). This study took place in Amhara region and aimed to evaluate the linkage between present herd management practices and prevalence of lamb mortality, as well as pinpointing out which health interventions to focus on in order to increase lamb survival.

### Occurrence of lamb mortality and connection to single practices

This study describes occurrence of lamb mortality between late 2018 and late 2019. During this period, the lamb mortality rate of 8% was notably lower than previous studies have presented (Fentie et al. 2016; Ayele and Urge 2018). Based on information from Getachew et al. (2015) and Fentie et al. (2016), speculative reasons for the low mortality found in the present study could be a low prevalence of disease and sufficient feed availability during the study period. During the interviews, there were questions covering both occurrence of disease outbreak in the herd and occurrence of feed shortage during the past year, but none of these was significantly associated with lamb mortality. Some households did, however, informally state that “last year there were no feed shortage, however, the year before there was”, as the year 2017–2018 was very dry according to farmers and local members of the research team.

Ten out of 273 individual practices were found to be significantly associated with lamb mortality (Table 3). When interpreting these, one should consider that with such a high quantity of variables, it is possible that a low number of explanatory variables will be significant by chance. Even so, practices that are deemed beneficial for lamb survival—e.g. regrouping of pregnant sheep and monitoring and assisting with colostral intake—are positively associated with lamb survival. In contrary, not restricting contact between own sheep and other sheep is accounted for as unfavourable for flock health and lamb survival and, indeed, negatively associated with lamb survival according to the results. This follows previous recommendations based on studies stressing the importance of these factors, e.g. to regroup during pregnancy (Holmøy et al. 2012), to use protective wear during lambing assistance (Rutherford et al. 2015), and the importance of colostrum intake (Nowak and Poindron 2006; Banchero et al. 2015). Another reflection of the variables in Table 3 is that some are linked in multi-level questions, or part of checkbox questions (e.g. farmer regroups the sheep when the ewe is known to be pregnant, or farmer guides the lamb to the teat in the event that mismothering occurs), and, hence, might be difficult to evaluate outside of its context. Conclusively, these correlations must be interpreted with caution.

### Occurrence of lamb mortality and connection to combination of practices

The score system was developed to evaluate if the combined effect of multiple executed desired practices is associated with a lower occurrence of lamb mortality. The results show that a household performing more desired practices had a significantly lower occurrence of lamb mortality in his/her herd. To identify combinations of desired practices that stood out in the data set, the score was sectioned into phases of the reproductive cycle of the sheep. Again, a higher amount of desired practices was found to be a significant protective factor against lamb mortality during the two phases (pregnancy and time around lambing). These findings, therefore, suggest that targeting interventions on management practices during pregnancy and during lambing likely are most effective to lower occurrence of lamb mortality.

### Practices incorporated during pregnancy phase and suggested interventions

This phase of the sheep reproductive cycle incorporated information regarding detection of pregnancy in the ewes, the specific management practices that were performed to support the pregnant ewes, and when during the pregnancy these practices were carried out. All households claimed to be able to identify if their ewes were pregnant, but 77.7% of them stated signs of pregnancy that are only visible during later pregnancy (> 98 days)—e.g. gets an enlarged udder and/or gets a round belly. However, if the pregnancy is detected and managed from an early stage (< 30 days), lamb survival rates, birth weight, and productivity throughout life increase (Sargison and Scott 2010; Fthenakis et al. 2012).

Pregnancy scanning by ultrasonography is considered an accurate method for both determining early pregnancy and number of lambs in the womb (Fridlund et al. 2013; Rekik 2017). As all households in the current study stated that the ewes very seldom carry more than one lamb, in combination with the lack of pregnancy scanning as a prevailing practice in the Ethiopian highlands, ultrasound should probably not be considered as the first choice method for this purpose today. Instead, close monitoring of mating occurrence (including repeated breeding) is recommended for accurate determination of impregnation, date of lambing as well as recording of barren ewes. This does demand separation of ewe(s) and ram unless under close supervision to avoid unwanted and non-registered mating.

In large groups, one might also consider a restricted mating period, to steer lambing into seasons of the year rich in feed, as well as more easily oversee mating records, feed management during pregnancy and lambing. This matter was incorporated in the mating phase; however, conformity was often lacking between farmer’s answers and interviewer’s observations.

Presupposed pregnancy detection is well managed, one can advance the care of the pregnant ewe(s), focusing on sufficient and well-balanced feed. Fthenakis et al. (2012) highlight the importance of this from early pregnancy (for placental growth) throughout pregnancy until lambing (for lamb growth), and Mukasa-Mugerwa et al. (1994) discuss extra feed during, especially late, pregnancy to increase lamb birth weight and viability. As previously mentioned in this paper, feed availability is scarce at times in the studied region, which further highlights the importance of feed supplementation. This is especially important as sheep keeping in the study area often incorporates communal pasture, with no guarantees that the ewes get sufficient nutritional quantity or quality. One of the two main reported causes of death of the lambs in this study was general weakness. An impaired general condition of the lambs at birth does in turn lead to a higher risk of starvation, as the lamb is too weak to suckle. It has been proven that a lack of feed—both in overall energy and in macronutrients—given to the mother during the last months of pregnancy directly affects the viability of the offspring at birth (De et al. 2019).

### Practices incorporated during time around lambing phase and suggested interventions

This phase of the sheep reproductive cycle incorporated information regarding household’s practices executed around lambing and included extra supervision of the pregnant ewes around the time of lambing and separating the ewe from the herd for better supervision and reduced risk for the lamb. As previously discussed, supervision around the time of lambing demands specific knowledge of delivery date—further implying that there is a need for detailed supervision of mating and detection of pregnancy.

The time around lambing also incorporates households’ practices regarding assistance of lambing, including hygienic aspects and immediate supportive or sanitary actions taken directly after lambing (removing placenta, helping the lamb to dry, cleaning the place of birth, etc.). The results from this study indicate that lamb mortality is at its highest < 1 days of age, which is in line with other studies (Binns et al. 2002; Fentie et al. 2016), and further illuminates the vulnerability of the lamb around lambing. According to Holmøy et al. (2017), the most common causes of death of lambs < 1 day of age were asphyxia, trauma, congenital malformation, and bacterial infections in the gastrointestinal tract. Prolonged lambing may increase the risk of such injuries and disease; however, incorrect assistance at lambing may result in an injured ewe and/or lamb or less vigorous lambs leading to delayed suckling and/or risk of hypothermia (Darwish and Ashmawy 2011; Jacobson et al. 2020). Inarticulate, general guidelines regarding lambing assistance are difficult to apply as the need, or type, of assistance is situation dependent.

To learn the objectives for when to assist and what to do in each situation, a lambing course for farmers to partake in could be an option. Hygienic aspects should be incorporated in favour of both sheep and human health (Gebretensay et al. 2019). A similar approach has been applied by the Swedish farm animal expert advisory organisation, Farm and Animal Health, with great success according to both veterinarians and farmers (U. König, personal communication, 2021). An adapted version would be feasible to apply also in Amhara, e.g. via ILRI-organised community conversations. These community conversations have previously been perceived well by the farmers, successfully spreading information and changing attitudes regarding sheep keeping (Lemma et al. 2021).

### Other factors linked to pregnancy and time around lambing

Colostrum intake is a crucial part of neonate lamb health and survival (Nowak and Poindron 2006; Banchero et al. 2015). In this study, however, colostrum intake was not incorporated into the phase time around lambing, but in phase neonatal lamb life (< 7 days), and was not significantly associated with lamb mortality. It can nevertheless be noted that all but one household stated that the lamb was allowed to suckle from the ewe from birth; hence, the importance of colostrum for the lamb is well known among the farmers.

### Differences between intervention and non-intervention villages

There was a significant difference in execution of desired practices between intervention and non-intervention villages during the phases mating period, pregnancy, and later lamb life. According to reports of the CGIAR Research Programme on Livestock (Haile et al. 2020; Haile and Getachew 2021), the intervention villages have implemented a breeding program based on the selection of rams for mating during the phase mating period and regular monitoring and weight follow-up of the lambs during phase later lamb life, which could explain the different outcomes. The reports do not explain why the practices during the phase pregnancy differ between these villages. Overall, results from the present study indicate that application of preventive management practices does make a difference in terms of increased lamb survival.

## Conclusion

Most lamb deaths in the Menz area in Amhara region, Ethiopia, occur within 24 h of birth, illuminating the importance of supplementary feeding throughout pregnancy for more viable lambs at birth, as well as correct management around lambing to potentiate lamb health from the time of delivery. Preventive herd management interventions should focus on early detection of pregnancy as this is key to managing the ewe throughout pregnancy and lambing. Even though the results should be interpreted with care, they indicate where to focus future interventions.

## Supplementary Information

Below is the link to the electronic supplementary material.

Supplementary file1 (PDF 932 KB)

## Data Availability

The datasets generated and analysed during the current study can be retrieved from the Swedish National Data repository at 10.5878/arqm-vq14. The data was managed in Epi Info™ 7.2.3.1 software and, thereafter, analysed in Microsoft Excel and the statistical software R (R Core Team 2020).
