# Supplementary material for: Preventive herd management practices and their effect on lamb mortality in Ethiopia
Source: Trop Anim Health Prod. 2023 Jan 19;55(1):42. doi: 10.1007/s11250-022-03361-x (PMC9852112; doi:10.1007/s11250-022-03361-x)
Supplement: Supplementary file 1 — Supplementary file1 (PDF 932 KB) [file 11250_2022_3361_MOESM1_ESM.pdf]

Preventive Herd Management Practices and Their Effect on Lamb Mortality in Ethiopia

E. Genfors[a]*, U. Magnusson[a], M. M. Moliso[b], B. Wieland[b], U. König[c], G. S. Hallenberg[a] & R. Båge[a]

[a]Department of Clinical Sciences, Faculty of Veterinary Medicine and Animal Science, Swedish University of Agriculture Agricultural University (SLU), Uppsala, Sweden

[b]International Livestock Research Institute (ILRI), Addis Ababa, Ethiopia

[c]Farm and Animal Health Organisation, Uppsala, Sweden

Dr. Wieland's current affiliation: Institute of Virology and Immunology, Mittelhaeusern, Switzerland &

Department of Infectious Diseases and Pathobiology, Vetsuisse Faculty, University of Bern, Bern, Switzerland

Dr Hallenberg's current affiliation: Public Health Agency of Sweden, Stockholm, Sweden

SLU, Department of Clinical Sciences, Box 7054, 750 07 Uppsala, Sweden

*Corresponding author: elisabeth.genfors@slu.se

Small Ruminant Herd Health

CaseID

Region

Date

Village

GPS coordinates

Intervention village

About the owner:

Name

Gender

Number of rams/ewes/lambs in the flock today:

Adult rams:

Adult ewes:

Lambs (<6 months), both genders:

1.1 Who is the main caretaker of the sheep?

1.2 How long have you been keeping sheep? (years)

1.3 How did you learn about sheep keeping?

- ☐ Family
- ☐ Friends
- ☐ Self-taught
- ☐ People in the village
- ☐ Development agents
- ☐ Research Institutes
- ☐ Other Please specify:

1.4 Have you ever received information or recommendations about sheep keeping or sheep health from authorities or animal health organizations?

1.4.1 If yes, have you been able to apply this to your herd?

1.4.1.1 If no, why not? Please describe:

1.5 Did you go to school?

1.5.1 If yes, what is the highest level of education do you have?

- ◯ Started elementary school did not finish (1-4)
- ◯ Full elementary school education (1-8)
- ◯ Junior secondary school (9-10)
- ◯ Senior secondary school (11-12)
- ◯ Further educated (College diploma and/or University degree)
- ◯ Other Please specify

2 HERD
(Definition herd: the group of sheep belonging to one specific sheep owner or caretaker)

2.1 Do you in any way restrict contact between your own sheep herd and other sheep?
(including: neighbour's sheep, sheep in village, sheep in markets, etc)

Comment:

2.2 During the past year, have you had any recurrent health issues in your herd?
(Definition: more than one sheep suffering from the same symptoms, eg coughing, GI-problems, unexpected deaths, etc.)

2.2.1 If yes, please specify symptoms:

2.3 What actions do you take if a sheep becomes sick within your herd? (tick more than one if necessary)

- ☐ Nothing
- ☐ I ask a person educated within veterinary medicine for advice/help *
- ☐ I ask someone else for advice/help
- ☐ I treat it, based on my own knowledge, with traditional medicines
- ☐ I treat it, based on my own knowledge, with medication I have bought from a drug store
- ☐ Other Please specify:

** IF KNOWN, please specify what profession this person has (animal health worker, DVM, etc)*

2.4 Do you regularly have your animals treated with medication of any sort also when they are healthy? (e.g. vaccinations, de-worming, preventive medication)

If yes:

2.4.1. What do you treat against?

2.4.2 How often?

2.4.3 Any specific treatment during the gestation period?

2.4.4 From where do you get the medication?

2.5 Have you encountered any critical feed shortage during the past year?

2.5.1 If yes, during which month(s)?

3. LAMBS IN THE HERD (definition lamb: ovine <6 months)

3.1 How many lambs are (in general) born by one ewe after one pregnancy?

3.2 How many lambs have been born in your herd the past year?

3.3 How many lamb deaths have you had during the past year? (if none, put 0)

If any lamb deaths:

3.3.1 Is there an age where lamb deaths were more frequent?

3.3.1.1 If yes, what age is that?

3.3.2.1 Among those deaths, how many lambs were born as twins?

3.3.2.2 Triplets?

3.3.2 What has been the cause(s) of death? (Disease, miss-mothering, cold, predators, delivery problems, etc.). Please describe:

3.4 In which season/month of the year does most of the lamb deaths occur?

3.5 Have you had any abortions during the past year?

3.5.1 If yes, how many?

3.6 Do you perceive lamb death as a problem within your herd?

3.7 Do you actively try to prevent lamb deaths?

3.7.1 If yes, how? Please describe

4. MATING

4.1 Do you actively plan the mating of your ewes?

If no go to question 4.2.

4.1.1 Do you intentionally try to increase of number of lambs? (flushing)

4.1.2 If you have multiple ewes: do you try to synchronize them to be able to mate them concurrently?

4.1.2.1 Why? Describe:

4.1.3 Do you consider what time of year you mate your ewe?

4.1.4 How do you decide when it is time to present your ewe to the ram? Describe:

4.2 Do you ever avoid mating one or more of your ewes?

[ ]

4.2.1 If yes, what could be reasons to avoid mating her/them? (tick all that apply)

- ☐ Because she is too old
- ☐ Because she is too young
- ☐ Because of her bad mothering abilities
- ☐ Because her health is not good enough (e.g. mastitis, general disease, etc)
- ☐ Because she is too thin
- ☐ Because she has had abortions before
- ☐ Because her last delivery was difficult
- ☐ Other Please specify: ____________

4.3. Do you check the body condition of your ewe before she is allowed to mate?

[ ]

4.3.1 If yes, please describe how:

____

4.4 Do you choose which ram is let to mate your ewe(s)?

[ ]

4.4.1 If yes, what ram do you use?

- ◯ A specific breeding ram/community ram (your own or someone else's)
- ◯ A specific ram because he has been good before (your own or someone else's)
- ◯ Any ram/no preference
- ◯ Artificial insemination
- ◯ Other please specify: ____________

4.5 Have you had, or do you have one or more ewes that does not get pregnant?

- ◯ Yes
- ◯ No
- ◯ I don't know

5. GESTATION PERIOD

5.1 Do you control if your ewe is pregnant?

[ ]

5.1.1 If yes, how?

- ◯ I check for signs (She doesn't mate with the ram anymore she gets a round belly or enlarged udder etc)*
- ◯ Ultrasound
- ◯ Blood tests
- ◯ Other   Please specify [ ]

Please specify what signs: [ ]

5.2 Do you know how many lambs your ewe is carrying before she delivers?

[ ]

5.2.1 If yes, how?

[ ]

5.3 Do you manage your ewe differently when she is assumed to be pregnant?

[ ]

5.3.1 If yes, how? (specify WHAT is done and WHEN during pregnancy it is done)

- ☐ I try to minimize stress
  Specify: [ ]
- ☐ I restrict contact with other sheep outside of my herd
  Specify: [ ]
- ☐ I re-group my sheep herd
  Specify: [ ]
- ☐ I change feeding routines
  Specify: [ ]
- ☐ I add supplements in her feed
  Specify: [ ]
- ☐ Other
  Specify: [ ]

5.4 Do you check the body condition of your ewe during pregnancy?

- ◯ Yes once
- ◯ Yes more than once
- ◯ No

6. DELIVERY

6.1 Do you know on beforehand when your ewe is expected to lamb?

- ◯ Yes vaguely (within a month)
- ◯ Yes more exactly (within a week)
- ◯ I don't know when she will lamb
- ◯ I don't know if she is pregnant
- ◯ Other Please specify: ____________

6.2 Do you give her extra care and/or supervision around the time of lambing? (tick all that apply)

- ☐ Yes, I look after her more often
- ☐ Yes, I separate her from the other sheep, if this has not been done before
- ☐ Yes, I shear the wool around her hind parts and udder
- ☐ Yes, other Please specify: ____________
- ☐ No

6.3 Do you monitor the lambing?

[▾]

6.3.1 If yes, do you assist during lambing?

- ◯ Yes always
- ◯ Yes if needed
- ◯ No

6.3.1.1 If yes, how? Please describe:

____

6.3.1.2 Do you wash your hands before and/or after helping out?

- ◯ Yes both before and after
- ◯ Yes after
- ◯ Yes before
- ◯ No

6.3.1.3 Are there any other hygiene procedures you consider at lambing? Please describe:

____

6.4 Where does your ewe give birth?

- ◯ Specific lambing pen
- ◯ Other type of shelter *
- ◯ Wherever the ewe happens to be at time of birth
- ◯ Other *

* Please specify: ____________________

6.5 At what time of the day does your ewe(s) most often give birth?

◯ Daytime ◯ Nighttime

6.6 Do you take any precautions directly after birth? (tick all that apply)

- ☐ Cleaning of the place of birth
- ☐ Removal of placental membranes
- ☐ Cleaning of the ewe (udder, hind parts, etc)
- ☐ Helping the lamb to dry
- ☐ Move the ewe and lamb to a new place
- ☐ Separate ewe and lamb from herd, if this has not been done earlier
- ☐ Other Please specify: ____________________

7. THE NEWLY DELIVERED EWE AND HER LAMB (definition: first seven days after delivery)

7.1 Does the lamb get to drink the first milk (colostrum) from the ewe?

[▾]

If no:

7.1.1 Why not?

____

If yes:

7.1.2 How long after birth does the lamb start to suckle, on average? (hours)

____

7.1.3 Do you ensure the lamb gets sufficient amount of colostrum?

[▾]

7.1.3.1 If yes, describe how:

____

7.1.4 If the lamb does not start to suckle or get insufficient amounts of colostrum, do you assist or supplement?

[▾]

If yes:

7.1.4.1 What do you give? ____________________

7.1.4.2 How often? ____________________

7.2 Do you examine the udder of the ewe?

- ◯ Yes always
- ◯ No never
- ◯ Only if I notice problems with the udder and/or suckling
- ◯ Other Please specify: [ ]

7.3 Do you make sure the ewe takes care of her lamb(s)? (mis-mothering)

[▾]

7.3.1 If she does not – what do you do? Please describe:

[ ]

7.4 Do you monitor the lambs' health in any other specific way during the first week? (e.g. make sure they feed? How do you control this? General health?)

[▾]

7.4.1 If yes, please describe: [ ]

7.5 Does the ewe get any specific or extra care during this period?

[▾]

7.5.1 If yes, please describe: [ ]

8. THE LAMB (definition: up to 6 months of age)

8.1 How are the lamb(s) weaned from their mother?

- ◯ Naturally
- ◯ Manual separation of ewe and lamb
- ◯ Other Please specify: [ ]

8.2 For how long does the lamb(s) suckle on average? (months)

[ ]

8.3 How old is the lamb when it is allowed to walk/graze with the rest of the herd? (weeks)

[ ]

8.4 Do you give your lambs supplements or supplementary feed? (including before and during weaning)

8.4.1 If yes, please specify what:

8.5 Do you continuously ensure your lambs gain weight?

8.5.1 If yes, how?

- ◯ Eye measurement
- ◯ Use of scale
- ◯ Other Please specify:

8.6 Do you de-worm your lambs?

8.6.1 If yes, at what age? (Months)

8.7 Do you vaccinate your lambs?

8.7.1 If yes, against which disease(s)?

8.6 Do you treat your HEALTHY lambs with any other specific medication (preventive medication)?

8.6.1 If yes, with what?
